# Comparison of LC-MS^3^ and LC-MRM Methods for Quantifying Amantadine and Its Application in Therapeutic Amantadine Monitoring in Human Plasma

**DOI:** 10.3390/molecules27217619

**Published:** 2022-11-07

**Authors:** Qiang Sun, Haiwei Cao, Yong Liu, Yanyan Li, Jing Huang

**Affiliations:** 1 Department of Laboratory Medicine, The First Hospital of Jilin University, Changchun 130061, China; 2 Genetic Diagnosis Center, The First Hospital of Jilin University, Changchun 130061, China

**Keywords:** LC-MS^3^, amantadine, therapeutic drug monitoring

## Abstract

A simple sample preprocessing method was developed for the quantitative determination of amantadine (AMT) in human plasma by liquid chromatography-tandem mass spectrometry cubed (LC-MS^3^). The LC-MS^3^ system comprised a Shimadzu Exion LC-20AD HPLC pump coupled with a QTRAP 5500 mass spectrometer. First, the plasma samples were pretreated using acetonitrile as the extracting solution to precipitate protein. Next, amantadine and amantadine-d_15_ (AMT-d_15_) were separated on an Agilent Poroshell 120 SB-C18 column (4.6 mm × 50 mm, 2.7 μm) using isocratic elution with solvent A (70% 0.1% formic acid) and solvent B (30% acetonitrile) at a flow rate of 0.8 mL/min. The total run time for each sample was 3 min. The system used triple-stage fragmentation transitions at *m/z* 152.2→135.3→107.4 for AMT quantification in the positive ion mode and *m/z* 167.0→150.3→118.1 for AMT-d_15_ quantification. The LC-MS^3^ assay was linear (r > 0.995) with a concentration range of 50–1500 ng/mL. The lower limit of quantification (LLOQ) was 50 ng/mL, and the intra-day and inter-day accuracies and precisions were less than 8.0% at all concentrations. In addition, the recoveries and matrix effect for AMT in human plasma were within acceptable limits. In terms of stability, AMT had no significant degradation under all conditions. All the results met the requirements of the guidelines of the Food and Drug Administration (FDA) for biological method validation. The novelty of the MS^3^ assay was that it presented a methodology with higher selectivity and sensitivity. This method was successfully applied to 44 human plasma samples, and the obtained quantitative results were compared with another liquid chromatography-multiple reaction monitoring (LC-MRM) method. The Passing-Bablok regression coefficients and Bland-Altman plot revealed no difference between the LC-MS^3^ and LC-MRM methods, implying that the developed LC-MS^3^ method is a reliable and accurate assay for AMT determination in human plasma. These results are also a proof of concept for determining chemicals in biological samples by the LC-MS^3^ strategy.

## 1. Introduction

**Figure 1:**
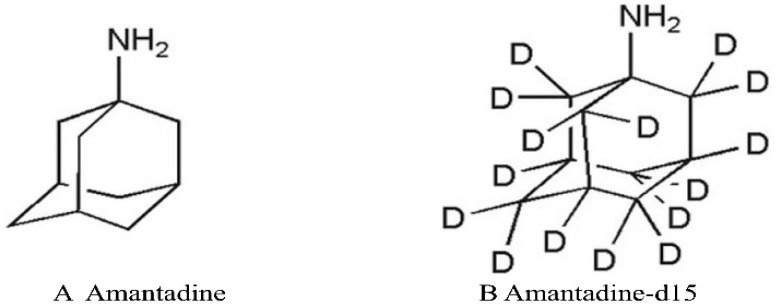
Chemical structure of Amantadine (**A**) and Amantadine-d_15_ (**B**).

Amantadine hydrochloride (C_10_H_17_N · HCl; Figure 1), also referred to as tricyclic [3.3.1.13.7] decane-1-amine) is a synthetic tricyclic amine of the adamantanes class. It is a derivative of adamantane that targets the M2 ion channel, primarily used for antiviral therapy. Amantadine (AMT) was approved by the Food and Drug Administration (FDA) in 2008 for clinical use in the treatment of Parkinson’s disease and multiple sclerosis [1,2]. In addition, AMT is used to treat extrapyramidal reactions caused by drugs, that act as non-competitive N-methyl-D-aspartate (NMDA) receptor antagonists, such as levodopa-induced dyskinesia (LID). Currently, AMT is the only drug with proven efficiency in alleviating LID [3,4,5,6]. 

AMT is a highly effective drug administered orally and mainly excreted by the kidney [7]. Clinical studies have revealed that patients with renal insufficiency might lead to an extended half-life for AMT [8,9]. However, according to clinical medication guideline recommendations, these patients and the elderly should monitor their AMT plasma concentrations. Generally, AMT causes severe side effects, including a dry mouth, lethargy, blurred vision, insomnia, consciousness disorder, and hallucinations, which are influenced by plasma AMT concentration and clinical dosage [10]. Therefore, AMT therapeutic drug monitoring (TDM) is necessary to maintain the therapeutic plasma concentration and avoid adverse reactions.

Currently, there are several TDM analytical methods for AMT, including immunoassays, which have been used for a long time [11,12], high-performance liquid chromatography with fluorescence detection (HPLC-Flu) [13,14], high-performance liquid chromatography combined with ultraviolet detection (HPLC-UV) [15], and liquid chromatography combined with mass spectrometry (LC-MS/MS) [16,17]. AMT has no particular UV absorption and fluorescence characteristics; thus, it requires derivatization by HPLC-UV and HPLC-Flu to enhance its sensitivity. However, compared to other analytical methods, LC-MS/MS assays are characterized by a simple sample processing technique, high sensitivity, and high selectivity. In addition, the LC-MS/MS methods have greatly improved precision and accuracy. As a result, LC-MS/MS assays are recommended as the gold standard for detecting compounds in biological samples. To date, quantitative detection of AMT by LC-MRM assays involves using QTRAP tandem mass spectrometers [18,19,20]. However, there are no reports on the detection of AMT in biological samples using triple-stage fragmentation (MS^3^) methods. Herein, we propose a method to improve the sensitivity and selectivity of AMT in quantitative detection using MS^3^ on a hybrid quadrupole-linear ion trap (QqLIT). We aimed to develop a highly sensitive and selective LC-MS^3^-based method to improve the quantitative detection of AMT in human plasma. In addition, we compared an LC-MRM-based method with the LC-MS^3^-based method. The MS^3^ detection is a scanning mode of QTRAP tandem mass spectrometry, with a high excitation efficiency and fast scanning rate (20,000 Da/s) [21,22]. During MS^3^ detection, the analyte precursor ions are first selected in Q1 and then fragmented in the collision cell (Q2) to generate product ions by collision-induced dissociation. Next, the product ions are enriched and captured by a linear ion trap (Q3) [23,24]. Finally, the selected product ions are further fragmented in the linear ion trap to generate secondary fragment ions detected by the detector. Overall, the MS^3^ scanning mode enhances selectivity and lowers the limit detection values using MRM^3^ transitions to achieve a more accurate quantification while removing interference and background noise.

To the best of our knowledge, this is the first attempt to quantify AMT in human plasma using an LC-MS^3^-based method.

## 2. Results and Discussion

### 2.1. Optimization of MS Conditions for Amantadine and Amantadine-d_15_

**Figure 2:**
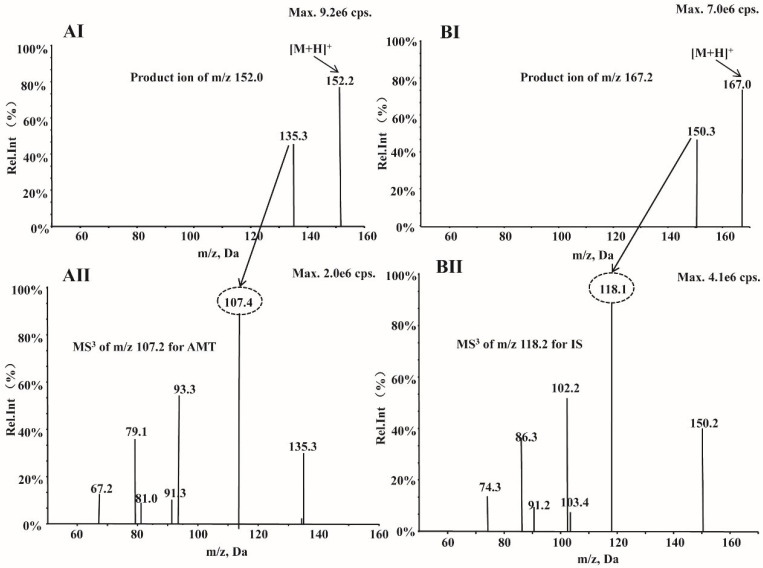
Product ion (MS^2^) and second-generation product ion (MS^3^) scans, respectively, for (**AI**) and (**AII**) AMT and (**BI**) and (**BII**) AMT-d_15_.

Both AMT and IS showed a better response in the positive ionization mode. The production (MS^2^) and second-generation product ion (MS^3^) spectra for AMT and IS are shown in Figure 2. The MS/MS transitions selected for detection of AMT and IS at MRM mode were at *m/z* 152.2→135.3 and 167.0→150.3, while in the MS^3^ mode, the MS/MS/MS transitions were at *m/z* 152.2→135.3→107.4 and 167.0→150.3→118.1, respectively. The optimized MS parameters for quantitation of AMT and IS are shown in Table 1. The mass range scanned for second-generation product ions of AMT was ±1.0 Da.

### 2.2. Optimization of LC Conditions

**Figure 3:**
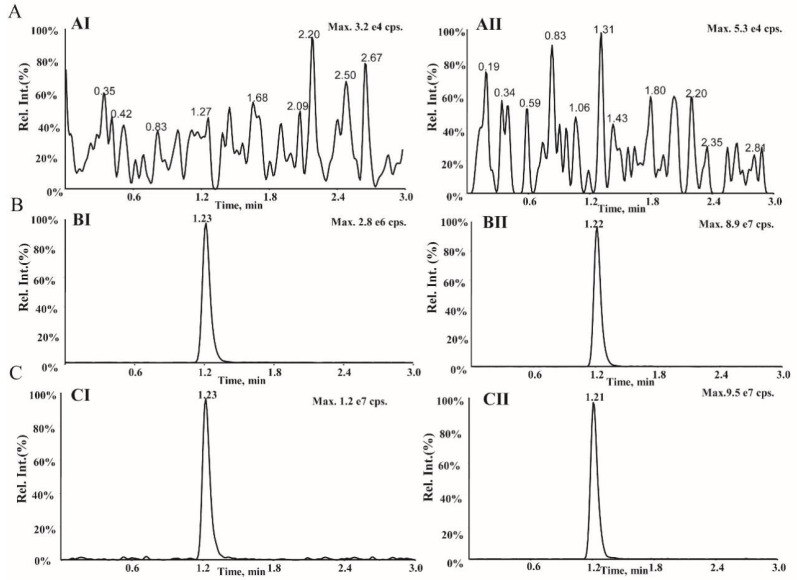
Representative LC-MS^3^ chromatograms of (**I**) AMT and (**II**) IS in (**A**) blank human plasma, (**B**) blank human plasma spiked with AMT at LLOQ (50 ng/mL) and IS (400 ng/mL), (**C**) actual plasma sample after oral administration of AMT.

An Agilent Poroshell 120 SB-C18 column (4.6 mm × 50 mm, 2.7 μm) using isocratic elution with 0.1% formic acid in water: acetonitrile (70:30, *v*:*v*) at a flow rate of 0.8 mL/min employed for chromatography separation gave symmetric peak shapes, adequate retention behaviors, and satisfactory mass spectrometric responses of AMT and IS. Under the optimum conditions, the retention times of AMT and IS were 1.23 and 1.21 min, respectively (Figure 3**)**.

### 2.3. Optimization of Sample Processing

Based on its high rapidity and simplicity, protein precipitation with acetonitrile was selected for sample processing. A total of 10 µL of plasma was mixed with 20 µL IS working solution and 1 mL acetonitrile to precipitate proteins. Protein precipitation with approximately 100 times dilutions yielded symmetrical peaks with a higher sensitivity above the matrix effect for AMT and IS. Therefore, protein precipitation using acetonitrile was selected in this study. LLOQ of 50 ng/mL is sufficient in this study. Furthermore, the LLOQ of this assay could easily be reduced by using more plasma, less dilution, or more injection volume.

### 2.4. Assay Validation

The representative MS^3^ chromatograms for AMT and IS in plasma are shown in Figure 3. The developed LC-MS^3^ assay revealed that there were no significant interferences at the retention time. The typical retention times were 1.23 and 1.21 min for AMT and IS, respectively. Besides, no enhancement in the responses of AMT and IS was observed in the blank plasma samples, suggesting negligible carryover (Figure 3A). Additionally, there was no cross-talk between MS channels between AMT and IS (Figure 3C). The LC-MS^3^ method was linear in 50–1500 ng/mL range, with a regression coefficient of r > 0.995. Intra- and inter-day precisions (relative standard deviation) were less than 10.7%, while the accuracy (relative error) ranged from 90.4 to 102.4% for four different concentrations, implying that the method was repeatable and reliable (Table 2). For the LC-MS^3^ method, the matrix effects at low, medium, and high QC nominal concentrations ranged from 99.0–102.9%, thus meeting the request of the assay (Table 3). The recoveries for three concentrations of AMT ranged from 97.2–98.2%, implying that the recoveries were reproducible and concordant across all the concentration ranges in this study (Table 3). Besides, the concentrations under the various test conditions had stabilities within ±15.0% of the nominal concentrations, implying no notable degradation of ATM under the storage conditions (Table 4).

### 2.5. Comparison between LC-MS^3^ and LC-MRM

**Figure 4:**
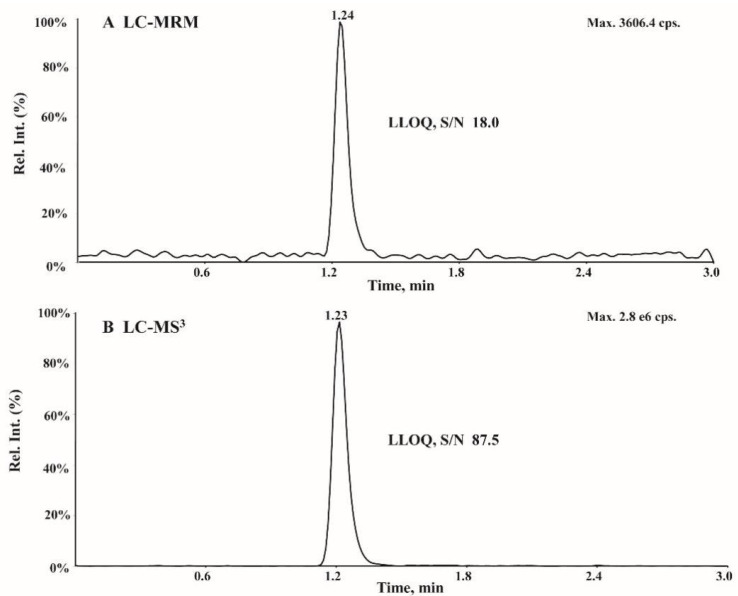
Representative chromatograms of AMT at the LLOQ (50 ng/mL) analyzed by (**A**) LC-MRM and (**B**) LC-MS^3^.

The LC-MRM method using MRM transitions at *m/z* 152.2→135.3 for AMT and *m/z* 167.0→150.3 for AMT-d_15_ was compared to the LC-MS^3^ method. The chromatogram of AMT at 50 ng/mL obtained from the LC-MS^3^ and LC-MRM methods is shown in Figure 4. The response signal of AMT using MRM acquisition was 3606 cps, while the S/N was 18.0 (Figure 4A). For MS^3^ acquisition, the response signal of AMT at 50 ng/mL was 2.8 e^6^ cps with an S/N of 87.5 (Figure 4B). The MS^3^ scan mode reduced the matrix interference and background noise via an additional fragmentation step; hence, the MS^3^ scan displayed a higher sensitivity than the MRM transition.

### 2.6. The Novelty and Significance of the LC-MS^3^ Method

The MS^3^ technique is restricted to QTRAP MS systems and ion trap MS systems. Herein, the LC-MS/MS system comprised of an HPLC with a QTRAP hybrid linear ion trap triple quadrupole mass spectrometer. The merits of the proposed LC-MS^3^ method include the high selectivity, high sensitivity, high signal to noise, high throughput (3 min per sample), and small sample volume (only 10 µL). To our knowledge, this is the first report on the use of the LC-MS^3^ technique for quantifying AMT and its application in therapeutic drug monitoring in human plasma. This study provides an innovative and promising alternative technique to the traditional LC-MRM technique, given the high sensitivity and high selectivity of the LC-MS^3^ technique.

### 2.7. Method Application

**Figure 5:**
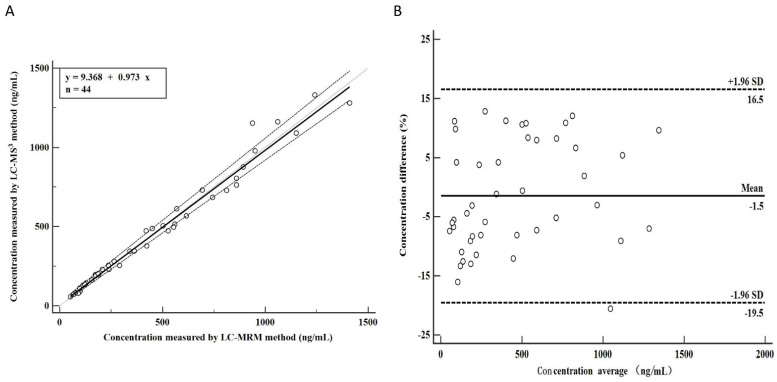
Comparison of the LC-MRM and LC-MS^3^ methods. (**A**) Passing-Bablok correlation plots of the concentration of AMT from clinical patients. The solid black lines indicate the Passing-Bablok regression. (**B**) Bland-Altman analysis verifies the difference in AMT concentration measured using the LC-MRM and the LC-MS^3^ in 44 human plasma samples.

Forty-four patients treated with AMT were selected to validate the clinical applicability of the LC-MS^3^ method. The AMT concentrations in 44 human plasma samples determined by the LC-MRM and the LC-MS^3^ methods are shown in Table S1. A comparison of the clinical applicability between the two methods is shown in Figure 5, which reveals that they were consistent with no constant and proportional deviation. A regression equation was generated by the Passing-Bablok analysis was y = 9.368 (95% CI,0.247,22.58) + 0.973 (95% CI,0.919,1.036)x(Figure 5A). A Bland-Altman plot further revealed the mean difference between the LC-MRM and the LC-MS^3^ method was −1.5% (95% LoA, −19.5% −16.5%), and the AMT differences were evenly distributed in both methods, with only one of the 44 samples exceeding the protocol limit of 1/44 (2.27%). The maximum concentration deviation of 97% AMT sample pairs was ±1.96SD (Figure 5B). Therefore, the LC-MRM and the LC-MS^3^ methods can be used for AMT drug monitoring without an obvious difference.

## 3. Materials and Methods

### 3.1. Chemical Reagents

AMT was acquired from Dr. Ehrenstorfer (Augsburg, Germany), while the internal standard (IS) AMT-d_15_ was acquired from Cambridge Isotope Laboratories (Andover, MA, USA). Formic acid (FA), acetonitrile, and methanol were purchased from Sigma Corporate (Billerica, MA, USA). The three reagents were HPLC grade. Ultrapure water was obtained from Watsons (Changchun, China).

### 3.2. Chromatographic and Mass Spectrometric Conditions

Liquid chromatographic analysis was carried out on a Shimadzu UFLC XR system equipped with two LC-20 AD XR binary pumps (pump A + B), SIL-20A XR AUTO sampler, and a CTO-20 AC column compartment. The injection volume was set at 1 µL. The column oven and auto-sampler were maintained at 40 and 25 °C, respectively. Chromatographic separation was performed on an Agilent Poroshell 120 SB-C18 column (4.6 mm × 50 mm, 2.7 μm) using isocratic elution with a mobile phase consisting of 70% 0.1% formic acid in water (phase A) and 30% acetonitrile (phase B). The flow rate was set at 0.8 mL/min.

Tandem mass spectrometry was performed on a QTRAP 5500 mass spectrometer (AB SCIEX, ON L4K 4V8, Canada) equipped with electrospray ionization in the positive ion mode. The linear ion trap (MS^3^) and multiple reaction monitoring (MRM) modes were used to quantify ATM and AMT-d_15_ (IS), respectively. The MRM^3^ transitions for ATM and IS identification in the MS^3^ mode were set at *m/z* 152.2→135.3→107.4 and 167.0→150.3→118.1, respectively. In the MRM mode, the MS/MS transitions were set at *m/z* 152.2→135.3 and 167.0→150.3, respectively. The optimized MS parameters for quantitative analysis of ATM and AMT-d_15_ (IS) are summarized in Table 1. Data acquisition and processing were performed by the analysis 1.6.3 software.

### 3.3. Preparation of Calibration Standards and Quality Control Samples

The AMT stock solution was prepared by dissolving 1 mg of AMT in 1 mL of a methanol: water (50:50, *v*/*v*) ratio. Next, the blank plasma was serially diluted in the stock solution to a final concentration of 50, 100, 300, 500, 700, 1000, and 1500 ng/mL, which were used as the calibration standards. Similarly, quality control (QC) samples of AMT at low (150 ng/mL), medium (600 ng/mL), and high (1200 ng/mL) concentrations were prepared. An IS stock solution of AMT-d_15_ was prepared in methanol and diluted to 400 ng/mL with an aqueous methanol solution consisting of a 50:50 (*v*/*v*) methanol to water ratio.

### 3.4. Plasma Sample Preparation

Frozen human plasma samples were thawed at room temperature before analysis. Next, 10 μL of plasma was added to 20 μL of IS working solution (400 ng/mL) and 1 mL of acetonitrile to precipitate the proteins. The mixtures were vortexmixed for five min on a shaker (Scientific Industries, Bohemia, NY, USA), then centrifuged (Cence, H1650R) at 13,000 rpm for 5 min at 4 °C. After centrifugation, 1 μL of the supernatant was transferred into the autosampler vial and injected into the LC-MS system.

### 3.5. Method Validation

Method validation was carried out following the bioanalytical method validation guideline of the U.S. FDA [25,26]. The parameters validated were selectivity, linearity, the lower limit of quantification (LLOQ), extraction recovery, matrix effect, accuracy, precision, stability, and dilution integrity. The protocol for assay validation is detailed in the supporting information.

### 3.6. Clinical Application

To verify the applicability of the LC-MS^3^ method, plasma samples from 44 patients were obtained from the rehabilitation department of the First Hospital of Jilin University. Samples were analyzed and the AMT levels were quantified. The plasma samples were anticoagulated with EDTA-2K, then centrifuged and frozen at −20 °C until samples were ready for preparation. The protocols in this study were reviewed and approved by the institutional ethics committee of the First Hospital of Jilin University (ethical approval number: 2021-522).

### 3.7. Statistical Analysis

Data acquisition, data processing, and graphic presentation were performed with Analyst 1.6.3 software (AB SCIEX, Foster City, CA, USA), Microsoft 2010 (Microsoft, Bellevue, DC, USA), and MedCalc Version 15.2.2 (MedCalc Software, Mariakerke, Belgium), respectively. Passing-Bablok regression and Bland-Altman analysis were adopted to analyze the agreement between the LC-MS^3^ and LC-MRM methods. The agreement was sufficient if the differences were within ±1.96 SD of the mean difference for ≥67% of the sample pairs [27,28]. Finally, the ATM plasma concentration was calculated based on the MRM and MS^3^ methods.

## 4. Conclusions

A simple, highly sensitive, selective, and high-throughput LC-MS^3^ assay was developed and validated in the present study for the quantitative analysis of AMT in the plasma of patients. The application of the LC-MS^3^ assay was tested on clinical samples and confirmed to be accurate and reliable; hence, it can be successfully applied in therapeutic drug monitoring. This work is a proof-of-concept for using the LC-MS^3^ technique for quantitative analysis of compounds in biological samples.

## Figures and Tables

**Table 1:** Optimized parameters for the quantitation of AMT using LC-MS^3^.

Parameters	MS^3^	
	Amantadine	Amantadine-d_15_(IS)
MS^3^ transitions	152.2→135.3→107.4	167.0→150.3→118.1
Declustering potential (V)	43	43
Entrance potential (V)	10	10
Collision energy (eV)	25	25
Excitation energy (AF_2_) (V)	0.1	0.1
Scan rate (Da/s)	10,000	10,000
LIT fill time (ms)	80	80
Excitation time (ms)	25	25
Turboheater temperature (°C)	450	450
Ionspray voltage (V)	5500	5500
Curtain gas (N_2_,psi)	15	15
Nebulizer gas (N_2_,psi)	50	50
Heater gas (N_2_,psi)	50	50

**Table 2:** Precision and accuracy for AMT in human plasma.

Compound	Spiked Concentration (ng/mL)	Intra-Day (n = 6)		Inter-Day (n = 18)	
		Accuracy (%)	Precision (CV, %)	Accuracy (%)	Precision (CV, %)
AMT	50	90.4 ± 9.5	10.6	94.5 ± 10.1	10.7
	150	93.9 ± 5.0	5.3	94.0 ± 7.8	8.3
	600	99.6 ± 4.0	4.1	102.4 ± 2.8	2.7
	1200	97.1 ± 4.2	4.3	98.5 ± 4.5	4.5

**Table 3:** Extraction recoveries and matrix effects of AMT in human plasma (n= 6).

Compound	Spiked Concentration (ng/mL)	Recovery (%)		Matrix Effect (%)	
		Average	CV	Average	CV
AMT	150	97.5 ± 10.5	10.7	100.8 ± 6.6	6.5
	600	98.2 ± 7.6	7.7	99.0 ± 8.8	8.9
	1200	97.2 ± 3.6	3.7	102.9 ± 4.9	4.0

**Table 4:** Stability of AMT in plasma and processed samples under various storage conditions (n = 3).

Compound	Spiked Concentration (ng/mL)	Bench-Top (3 h, RT)	Processed Auto-Sampler (24 h, RT)	Freeze-Thaw (−20 °C)	Long-Term (4 W, −80 °C)
AMT	150	90.7 ± 6.7	104.3 ± 3.5	104.3 ± 5.8	106.1 ± 3.8
	1200	93.6 ± 7.9	91.7 ± 4.3	97.5 ± 1.7	99.5 ± 4.9

Abbreviation: RT, room temperature.

## Data Availability

The data presented in this study are available upon request from the corresponding author.

## Supplementary Materials

The following supporting information can be downloaded at: https://www.mdpi.com/article/10.3390/molecules27217619/s1; Table S1 Concentrations of amantadine in 44 human plasma samples analyzed by LC-MRM and LC-MS^3^.

## Sample Availability

Samples of the compounds are available from the authors.

## References

[1] Stiver G. (2003). The treatment of influenza with antiviral drugs. CMAJ.

[2] Leonov H., Astrahan P., Krugliak M., Arkin I.T. (2011). How do aminoadamantanes block the influenza M2 channel, and how does resistance develop?. J. Am. Chem. Soc..

[3] Brigham E.F., Johnston T.H., Brown C., Holt J.D.S., Fox S.H., Hill M.P., Howson P.A., Brotchie J.M., Nguyen J.T. (2018). Pharmacokinetic/Pharmacodynamic Correlation Analysis of Amantadine for Levodopa-Induced Dyskinesia. J. Pharmacol. Exp. Ther..

[4] Wang C.C., Wu T.L., Lin F.J., Tai C.H., Lin C.H., Wu R.M. (2022). Amantadine treatment and delayed onset of levodopa-induced dyski-nesia in patients with early Parkinson’s disease. Eur. J. Neurol..

[5] Romrell J., Fernandez H.H., Okun M.S. (2003). Rationale for current therapies in Parkinson’s disease. Expert. Opin. Pharmacother..

[6] Deep P., Dagher A., Sadikot A., Gjedde A., Cumming P. (1999). Stimulation of dopa decarboxylase activity in striatum of healthy human brain secondary to NMDA receptor antagonism with a low dose of amantadine. Synapse.

[7] Bleidner W.E., Harmon J.B., Hewes W.E., Lynes T.E., Hermann E.C. (1965). Absorption, distribution and excretion of amantadine hydrochloride. J. Pharmacol. Exp. Ther..

[8] Aoki F.Y., Sitar D.S. (1988). Clinical pharmacokinetics of amantadine hydrochloride. Clin. Pharmacokinet..

[9] Horadam V.W., Sharp J.G., Smilack J.D., McAnalley B.H., Garriott J.C., Stephens M.K., Prati R.C., Brater D.C. (1981). Pharmacokinetics of amantadine hydrochloride in subjects with normal and impaired renal function. Ann. Intern. Med..

[10] deVries T., Dentiste A., Di Lea C., Pichette V., Jacobs D. (2019). Effects of Renal Impairment on the Pharmacokinetics of Once-Daily Amantadine Extended-Release Tablets. CNS Drugs.

[11] Yu W., Zhang T., Ma M., Chen C., Liang X., Wen K., Wang Z., Shen J. (2018). Highly sensitive visual detection of amantadine residues in poultry at the ppb level: A colorimetric immunoassay based on a Fenton reaction and gold nanoparticles aggregation. Anal. Chim. Acta..

[12] Xie S., Wen K., Wang S., Wang J., Peng T., Mari G.M., Li J., Wang Z., Yu X., Jiang H. (2019). Quantitative and rapid detection of amantadine and chloramphenicol based on various quantum dots with the same excitations. Anal. Bioanal. Chem..

[13] Puente B., Hernandez E., Perez S., Pablo L., Prieto E., Garcia M.A., Bregante M.A. (2011). Determination of memantine in plasma and vitreous humour by HPLC with precolumn derivatization and fluorescence detection. J. Chromatogr. Sci..

[14] Higashi Y., Uemori I., Fujii Y. (2005). Simultaneous determination of amantadine and rimantadine by HPLC in rat plasma with pre-column derivatization and fluorescence detection for pharmacokinetic studies. Biomed. Chromatogr..

[15] Michail K., Daabees H., Beltagy Y., Elkhalek M.A., Khamis M. (2013). High-performance liquid chromatographic determination of memantine in human urine following solid-phase extraction and precolumn derivatization. J. AOAC Int..

[16] Bhadoriya A., Rathnam S., Dasandi B., Parmar D., Sanyal M., Shrivastav P.S. (2018). Sensitive and rapid determination of amantadine without derivatization in human plasma by LC-MS/MS for a bioequivalence study. J. Pharm. Anal..

[17] Maksymiuk A.W., Tappia P.S., Bux R.A., Moyer D., Huang G., Joubert P., Miller D.W., Ramjiawan B., Sitar D.S. (2021). Use of amantadine in the evaluation of response to chemotherapy in lung cancer: A pilot study. Future. Sci. OA.

[18] Jiang J.G., Zhang X.R., Zhang Y.H., Song G.S. (2013). Rapid identification 15 effective components of anti common cold medicine with MRM by LC-MS/MS. Acta Pharm. Sin..

[19] Liu Z., Yang F., Yu K., Lin Y., Liu S., Zhang Q., Su Z. (2012). Multi-residue determination of five antiviral drugs in chicken tissues by liquid chromatography-electrospray ionization tandem mass spectrometry. Chin. J. Chromatogr..

[20] Yun H., Cui F., Yan H., Liu X., He Y., Zhang Z. (2013). Determination of ribavirin and amantadine in chicken by ultra performance liquid chromatography-tandem mass spectrometry. Chin. J. Chromatogr..

[21] Dziadosz M., Klintschar M., Teske J. (2017). Imatinib quantification in human serum with LC-MS3 as an effective way of protein kinase inhibitor analysis in biological matrices. Drug Metab. Pers. Ther..

[22] Sanda M., Pompach P., Benicky J., Goldman R. (2013). LC-MS3 quantification of O-glycopeptides in human serum. Electrophoresis.

[23] Kobayashi H., Imai K. (2021). Recent Progress in FD-LC-MS/MS Proteomics Method. Front. Chem..

[24] Seger C., Salzmann L. (2020). After another decade: LC-MS/MS became routine in clinical diagnostics. Clin. Biochem..

[25] Okhina A.A., Rogachev A.D., Yarovaya O.I., Khvostov M.V., Tolstikova T.G., Pokrovsky A.G., Khazanov V.A., Salakhutdinov N.F. (2020). Development and validation of an LC-MS/MS method for the quantitative analysis of the anti-influenza agent camphecene in rat plasma and its application to study the blood-to-plasma distribution of the agent. J. Pharm. Biomed. Anal..

[26] Arndt T., Guessregen B., Hohl A., Reis J. (2005). Determination of serum amantadine by liquid chromatography-tandem mass spectrometry. Clin. Chim. Acta..

[27] van Nuland M., Rosing H., Schellens J.H.M., Beijnen J.H. (2020). Bioanalytical LC-MS/MS validation of therapeutic drug monitoring assays in oncology. Biomed. Chromatogr..

[28] Van Eeckhaut A., Lanckmans K., Sarre S., Smolders I., Michotte Y. (2009). Validation of bioanalytical LC-MS/MS assays: Evaluation of matrix effects. J. Chromatogr. B Analyt. Technol. Biomed. Life Sci..

