# Supplementary material for: Comparison of LC-MS3 and LC-MRM Methods for Quantifying Amantadine and Its Application in Therapeutic Amantadine Monitoring in Human Plasma
Source: Molecules. 2022 Nov 7;27(21):7619. doi: 10.3390/molecules27217619 (PMC9655109; doi:10.3390/molecules27217619)
Supplement: Supplementary file 1 [file molecules-27-07619-s001.zip › molecules-1954834-supplementary.pdf]

Supplemental Materials

Method validation

Selectivity, linearity and lower limit of quantification

Selectivity was evaluated by analyzing the plasma and blood samples from six healthy volunteers. Selectivity was also investigated by spiking blank samples with the IS or a standard at the lower limit of quantitation (LLOQ) to check the signals in the channel of the amantadine. An interference of <20% of the LLOQ level (<5% for the IS) at the observed retention time(RT) window of the analyte (30 s) was acceptable for selectivity.

The linearity was examined by analyzing calibration standards in plasma samples at concentration ranging from 50-1500 ng/mL on three non-consecutive days. Calibration curves were constructed by plotting peak area ratio (y) of MER/IS versus its nominal concentration (x) using a 1/x2 weighting factor. An acceptable determination coefficient (r> 0.995) was obtained. The LLOQ was defined as the lowest concentration of the calibration curve. The reproducibility at the LLOQ level was evaluated by consecutively injecting six processed samples at the LLOQ level and comparing its precision and accuracy against the established linearity. A maximum variation of ≤20% was observed.

Precision and accuracy

Precision (coefficient of variation, CV or relative standard deviation, RSD) and accuracy (relative error, RE) are assayed by analyzing six sets of replicates of LOQ and QC samples. QC samples at different levels (50,150,600,1200 ng/mL)were evaluated in three batches to examine the intra- and inter-day precision and accuracy of the method. For intra-day analysis six replicates at each level of QC concentration within the batch were examined, whereas for the inter-day analysis, 18 replicates from all the batches were examined. The precision should not exceed 15% (20% for LOQ) of RSD and accuracy should be within 85-115% (80-120% for LOQ) of the nominal concentration.

Matrix effect and recovery

The extraction recovery and matrix effect of the method were determined by analyzing six samples at three different levels (150,600,1200 ng/mL). In this validation, three separate sets were prepared. Set A was made by spiking QC samples and IS (400 ng/mL) samples into blank plasma

samples. Set B was prepared by spiking the neat standard (QC working solutions) and IS samples at the same concentrations after the plasma extraction process (post-extraction addition). Set C was acquired by adding QC working solutions and IS into an equivalent volume of pure solutions(water). The extraction recovery and matrix effect can be examined by the following formula: recovery (%) = A/B × 100,and matrix effect (%) = B/C × 100.

Stability and dilution integrity

The stability was evaluated for amantadine in human plasma by analyzing QC samples with 150 and 1200 ng/mL at different storage conditions. Long term stability was tested for plasma samples after storage at -80◦C for 4 weeks. Short term stability was studied on analysis of QC samples after three freeze-thaw, and at room temperature (RT) for 3h. Processed samples stored at RT for 24 h were reanalyzed for auto sampler stability.At all conditions, a maximum loss of 15% of amantadine concentration was acceptable stability.

Table S1 Concentrations of amantadine in 44 human plasma samples analyzed by LC-MRM and LC-MS^3.

Sample ID	MRM	MS^3	%
sample1	693	730	94.9
sample2	240	231	103.9
sample3	52	56	92.9
sample4	362	347	104.3
sample5	615	568	108.3
sample6	860	805	106.8
sample7	893	876	101.9
sample8	1060	1161	91.3
sample9	1150	1090	105.5
sample10	127	144	88.2
sample11	743	684	108.6
sample12	265	281	94.3
sample13	812	728	111.5
sample14	1240	1130	93.2
sample15	527	474	111.2
sample16	290	255	113.7
sample17	560	515	108.7
sample18	206	231	89.2
sample19	78.8	83.3	94.6
sample20	173	197	87.8

sample21	94.9	86	110.3
sample22	236	256	92.2
sample23	423	378	111.9
sample24	340	344	88.6
sample25	569	612	93.0
sample26	860	762	112.9
sample27	420	474	88.6
sample28	502	505	99.4
sample29	99.1	95	104.3
sample30	938	1153	81.4
sample31	554	497	111.5
sample32	950	979	97.0
sample33	1410	1280	110.2
sample34	112	128	87.5
sample35	190	196	96.9
sample36	449	487	92.2
sample37	89.7	80.2	111.8
sample38	156	163	95.7
sample39	74.9	80.1	93.5
sample40	95.7	112.4	85.1
sample41	174	195.5	91.3
sample42	122	136.1	89.6
sample43	187	203.2	92.0
sample44	65.5	71.7	94.1
